# Antibiotic Treatment of *Acinetobacter baumannii* Superinfection in Patients With SARS-CoV-2 Infection Admitted to Intensive Care Unit: An Observational Retrospective Study

**DOI:** 10.3389/fmed.2022.910031

**Published:** 2022-06-03

**Authors:** Erika Casarotta, Elisa Bottari, Sara Vannicola, Rachele Giorgetti, Roberta Domizi, Andrea Carsetti, Elisa Damiani, Claudia Scorcella, Vincenzo Gabbanelli, Simona Pantanetti, Benedetto Marini, Abele Donati, Erica Adrario

**Affiliations:** ^1^Department of Biomedical Sciences and Public Health, Università Politecnica delle Marche, Ancona, Italy; ^2^Anesthesia and Intensive Care Unit, Azienda Ospedaliero-Universitaria “Ospedali Riuniti”, Ancona, Italy

**Keywords:** *Acinetobacter baumannii*, superinfection, SARS-CoV-2, acute respiratory failure, antibiotics

## Abstract

**Introduction:**

In COVID-19 patients on mechanical ventilation, VAP from *Acinetobacter baumannii* remains a crucial risk factor for death. Antibiotic resistance represents an important problem in treating this infection. This study aims to describe the evolution of the superinfection from *PDR Acinetobacter baumannii* in patients with acute respiratory failure from SARS-CoV-2 infection admitted to ICU and compare the impact of two different antibiotic strategies on microbiological negativization.

**Methods:**

*S*ingle-center observational retrospective study, including patients admitted to our ICU from March 2020 to May 2021 for acute respiratory failure from SARS-CoV-2 infection who developed *PDR Acinetobacter baumannii* superinfection. Clinical data at ICU admission were collected, as well as the timing of isolation of *Acinetobacter baumannii*, its resistance profile, the site of infection, and the antibiotic therapy.

**Results:**

Of the 32 patients enrolled, 10 patients (31.2%) were treated with the combination of high-dose ampicillin/sulbactam, high-dose tigecycline, intravenous and inhaled colistin *(Protocol)*, the other 22 (68.8%) were treated with the combination of two antibiotics *(Control)*. Of the 10 patients in the *Protocol* group, 8 patients (80%) received also fosfomycin. All patients (100%) in the *Protocol* group had microbiological negativization, while in the *Control* group microbiological negativization was observed in 8 (36.4%) patients, *p* < 0.01.

**Conclusion:**

Our report shows microbiological negativization in all patients treated with the combination therapy of nebulized and intravenous colistin, high-dose tigecycline, and high-dose ampicillin/sulbactam. This combination of antibiotics seems to be a useful alternative when other treatments are not available or fail.

## Introduction

In December 2019 new cases of pneumonia of unknown origin came to light in China (1). The new virus was recognized as a coronavirus able to cause the severe acute respiratory syndrome (2). Therefore, it was named SARS-CoV-2, and the pathology derived from it was called Coronavirus Disease 2019 (COVID-19) (2). It is widely known that the clinical presentation of the illness may vary considerably. In some cases, the disease may be asymptomatic, while 5–15% of patients may experience dyspnea and respiratory effort and require endotracheal intubation and mechanical ventilation (3, 4).

In the case of intubated patients in intensive care units (ICUs), Ventilator-Associated Pneumonia (VAP) remains a crucial risk factor for death (5). A VAP is diagnosed when new pneumonia is detected after 2 days from the patient being intubated and mechanically ventilated. As for causative agents, the most common pathogens include *Staphylococcus spp., Enterococcus spp., Klebsiella pneumonia, Enterobacter spp., Escherichia coli, Acinetobacter spp.*, and *Pseudomonas spp.* (6).

Furthermore, a significant percentage of patients, admitted to ICU, is treated with broad-spectrum antibiotics, which increase the risk of developing hospital-acquired infections, particularly from multi-drug resistant (MDR) pathogens. Among them, *Multi-Drug Resistant Acinetobacter baumannii* (MDR-AB) represents a causative agent for almost half of Ventilator-Associated Pneumonia (VAP) (7) and is a severe problem in patients with COVID-19 in ICU (8, 9).

The most important risk factors for VAP from *Acinetobacter baumannii* are high blood pressure, chronic obstructive pulmonary disease (COPD), length of stay in ICU, at least one organ failure, chronic renal impairment, and reduced blood oxygenation level. Interestingly, these features are usually common to COVID-19 patients in ICU, who, therefore, become highly susceptible to the infection (10–12). *Acinetobacter baumannii* is a Gram-negative bacterium, opportunistic, pleomorphic, and non-motile. It can colonize dry surfaces and devices surviving up to 33 days (13–15). Moreover, the pathogen can develop resistance to numerous classes of antibiotics more rapidly than other bacteria. Therefore, it has been considered a major health problem in the international medical community (16). In regards to antimicrobial therapy, in the case of a *Multi Drug-Resistant Acinetobacter baumannii*, carbapenems still represent the treatment of choice. Unfortunately, the resistance to carbapenems has increased making the pathogen *eXtensively Drug-Resistant* (XDR), while other strains have been named *Pan Drug-Resistant* (PDR) when they showed resistance to polymyxins, especially colistin, and tigecycline (17). As for XDR AB, one of the last options is colistin which is highly nephropathic and neurotoxic (18). However, by changing the way of administration, the risk of nervous and renal damage can be decreased. When colistin is given by inhalation, the systemic distribution of the drug is reduced (19). Therefore, nebulized colistin seems to be a reasonable choice in the case of *Carbapenem-Resistant Acinetobacter baumannii* in patients with COVID-19 in ICU (5).

The higher incidence of *Pan Drug-Resistant Acinetobacter baumannii* causing VAP is observed particularly in Greece, Spain, and Italy, implying the need for new therapeutic strategies (20).

Thus, some authors proposed to use of a combination of antibiotics to exploit the synergistic effect of different classes (21).

In 2019, *Assimakopoulos et al.* reported positive results in treating 10 ICU patients with VAP from *Pan Drug-Resistant Acinetobacter baumannii* with a combination of antibiotics, which consisted of a high dose of tigecycline and ampicillin/sulbactam, and colistin, given both by inhalation and intravenously (22). As for sulbactam, its use is justified by its intrinsic activity against several strains of AB (23, 24).

The present brief report aimed to describe retrospectively the evolution of the superinfection from *PDR Acinetobacter baumannii* in patients with SARS-CoV-2 infection admitted to ICU. In addition, it assessed the incidence of negativization between patients treated with the combination of at least three antibiotics, according to a treatment protocol applied in our ICU, and those who received a combination of two antibiotics.

## Materials and Methods

We retrospectively collected the data from adult patients admitted to a single COVID-ICU (Anesthesia and Intensive Care Unit, University Hospital “Ospedali Riuniti” of Ancona, Italy) for acute respiratory failure from SARS-CoV-2 infection and *Acinetobacter baumannii* superinfection. We collected demographic data, including age, sex, body mass index (BMI) and comorbidities, and clinical data at ICU admission among which respiratory parameters and blood tests including lymphocytes, leukocytes, and procalcitonin. We calculated the SOFA *(Sequential Organ Failure Assessment)* score and the *Charlson Comorbidity Index* at ICU admission. The immunomodulatory and immunosuppressive therapies, if administered before admission, were noted. Any microbiological tests performed at the beginning and during the stay in ICU were reviewed. We noted the date of positivity to SARS-CoV-2, detected with the reverse transcriptase-polymerase chain reaction (RT-PCR) on the nasopharyngeal swab, performed before ICU admission. We also noted the precise timing of isolation of *Acinetobacter baumannii*, its resistance profile (MDR, XDR, PDR), and the site of infection. *Acinetobacter baumannii* strains from all kinds of cultures were identified in our microbiology laboratory with the new-generation mass spectrometry microbial identification system, VITEK^®^ MS PRIME *(bioMérieux, Marcy-l’Étoile, France).* To test the antimicrobial susceptibility was used the VITEK^®^2 System *(bioMérieux, Marcy-l’Étoile, France)* for all antibiotics. The resistance to colistin detected with the VITEK^®^2 System was confirmed with the broth microdilution method. The results were interpreted following the latest EUCAST breakpoints for *Acinetobacter baumannii* spp. available.

Following the definition of the resistance profile (17), we considered as *Multi Drug-Resistant* (MDR) *Acinetobacter baumannii* resistant to at least three classes of antimicrobial agents (all penicillins and cephalosporins, including inhibitor combinations, fluoroquinolones, and aminoglycosides), *eXtensively Drug-Resistant (XDR)* the *MDR Acinetobacter baumannii* resistant also to carbapenems and *Pan Drug-Resistant (PDR)* the *XDR Acinetobacter baumannii* resistant to polymyxins and tigecycline. In addition, data regarding antimicrobial treatment were collected. Starting from the second wave of the pandemic, when the problem of *PDR Acinetobacter baumannii* superinfection became very consistent in COVID-19 patients admitted to our ICU, impacting the length of stay and the outcome, we started to apply a protocol of antibiotic therapy based on the case series study of *Assimakopoulos et al.* (22). Patients with *PDR Acinetobacter baumannii* superinfection received combination therapy with intravenous colistin at the loading dose of 9 million IU followed by a maintenance dose of 4.5 million IU every 12 h, intravenous tigecycline at the dose of 100 mg every 12 h, intravenous ampicillin/sulbactam, administered in a continuous infusion, at the dose of 12 gr per day and inhaled colistin at the dose of 3 million IU every 6 h, added in case of respiratory tract infection. Sometimes, in patients with particularly severe clinical conditions, we added also fosfomycin at the dose of 12 gr per day. The maintenance dose of intravenous colistin in patients with the impaired renal function was adjusted with the use of the colistin calculator, based on the pharmacokinetic modeling data published by *Garonzik et al.* (25). We also reduced the dose of ampicillin/sulbactam in patients with a creatinine clearance less than 30 ml/min, according to the Cockcroft-Gault equation. Considering that rapid molecular systems to detect the pathogen were not routinely used in the period of study, we used to start this combination of antibiotics about 48–72 h after the cultural tests, as soon as the microbiological examinations reports were made available. Before the application of this protocol of the three antibiotics in combination, patients with *PDR Acinetobacter baumannii* were treated with nebulized and intravenous colistin alone or combined with an antibiotic of another class. To define the resolution of the infection, we considered both the clinical improvement in the signs of infection and the laboratory or instrumental parameters and the negativization from *Acinetobacter baumannii* in control culture tests. We also reported the complications of antibiotic therapy. According to the KDIGO guidelines (26), we defined *Acute Kidney Injury (AKI)* as the presence of any of the following criteria: an increase in serum creatinine by ≥ 0.3 mg/dl (≥ 26.5 μmol/l) within 48 h or an increase in serum creatinine to ≥ 1.5 times baseline, which is known or presumed to have occurred within the prior 7 days, or urine volume < 0.5 ml/kg/h for 6 h.

### Statistical Analysis

The statistical analysis was performed using STATA 17.0 BE – Basic Edition *(StataCorp, Texas, United States).* Categorical data were expressed as absolute and relative frequencies, numerical data as mean ± standard deviation, if normally distributed, or median [interquartile range], if not normally distributed. Normality of distribution was assessed using the Shapiro-Wilk test. Dichotomous data were compared using the chi-square test or Fisher’s exact test, as appropriate. Continuous variables were compared using the Student’s *t*-test for unpaired data or the Wilcoxon rank-sum test, as appropriate. A *p* < 0.05 was used to indicate the statistical significance.

Given the descriptive nature of the primary objective, a sample size calculation was not necessary.

### Ethical Aspects

The study protocol was approved by the local Ethics Committee (Comitato Etico Regionale delle Marche). All the data were anonymously analyzed. Written informed consent was not applicable due to the retrospective nature of the study.

## Results

We considered 32 patients, admitted to our ICU from March 2020 to May 2021 for acute respiratory failure consequent to SARS-CoV-2 infection who developed *PDR Acinetobacter baumannii* superinfection. The MIC (Minimum Inhibitory Concentration) values of the *PDR Acinetobacter baumannii* in the study population are presented in Table 1. In 30 patients (93.7%) the site of *PDR Acinetobacter baumannii* superinfection was the respiratory tract, in 2 patients (6.3%) the microorganism was isolated firstly in the rectal swab and then also in the respiratory tract cultures. The median age of patients was 59.5 [54–66] years and 28 (87.5%) were males. Of the 32 patients, 10 patients (31.2%) were treated with the combination of high-dose ampicillin/sulbactam, high-dose tigecycline, intravenous and inhaled colistin *(Protocol)*, the other 22 (68.8%) were treated with the combination of two antibiotics *(Control)*. Of the 10 patients in the *Protocol* group, 8 patients (80%) received also fosfomycin. In all the 10 patients of the *Protocol* group, the *PDR Acinetobacter baumannii* was isolated only in the respiratory swab. The demographic and clinical characteristics of the two groups of patients are presented in Table 2.

**TABLE 1:** Minimum Inhibitory Concentration (MIC) values of the *PDR Acinetobacter baumannii* in the study population.

Antimicrobial agent	MIC values (mg/L)	MIC breakpoints (mg/L)* R >
Amikacin	≥ 64	8
Ciprofloxacin	≥ 4	1
Colistin	> 2	2
Gentamicin	≥ 16	4
Meropenem	≥ 16	8
Trimethoprim/Sulfamethoxazole	≥ 320	4
Tigecycline	2-4	–

*EUCAST Clinical breakpoints tables, v. 10.0 and 11.0.

*R = resistant; – = no EUCAST breakpoint available.*

**TABLE 2:** Demographic and clinical characteristics at Intensive Care Unit (ICU) admission of the two groups of patients.

Characteristics	Protocol (*n* = 10)	Control (*n* = 22)	p-value*
Male sex, n (%)	8 (80)	20 (90.9)	0.57
Age, years	58.5 [55–66]	60 [53–66]	0.97
BMI, kg/m^2^	33.5 [26–35.9]	27.8 [25.5–31.2]	0.12
Charlson Comorbidity Index, points	2.2 ± 2.2	2.1 ± 1.5	0.84
PaO_2_/FiO_2_	74.5 [63–82]	78.5 [61–87]	0.67
WBC, ×10^3/mm	12.1 [7.8–14]	11.8 [7.8–19.2]	0.81
Lymphocytes, ×10^3/mm	0.98 [0.63–1.24]	0.56 [0.4–0.69]	0.07
Procalcitonin, ng/ml	0.33 [0.17–0.93]	0.39 [0.11–1.03]	0.96
SOFA score	6.5 ± 1.6	7.7 ± 2.3	0.13

*Data are presented as absolute and relative frequencies, mean ± standard deviation, median [interquartile range].*

*Chi-squared test or Fisher’s exact test for categorical variables and Student’s t-test or Wilcoxon rank-sum test for numerical variables, as appropriate.

*BMI = body mass index; PaO_2_/FiO_2_ = ratio of partial pressure of arterial oxygen to fraction of inspired oxygen; WBC = white blood cells; SOFA = Sequential Organ Failure Assessment.*

Between the two groups of patients, no significant differences were observed in demographic and clinical characteristics at admission to the ICU. Considering the therapy received before the ICU admission, 12 patients (54.5%) in the *Control* group and 7 (70%) in the *Protocol* group had already been treated with antibiotics, *p* = 0.28. There was no significant difference in the duration of the steroid therapy received before the ICU admission [8 (2–11) days in the *Control* group vs. 4 (1–10) days in the *Protocol* group, *p* = 0.52]. The mean length of stay in the ICU of patients in the *Control* group was 25.2 ± 17.3 days, instead, for patients in the *Protocol* group was 36.1 ± 32.6 days, *p* = 0.36. All patients, 100% (95% CI: [69–100]%), in the *Protocol* group had microbiological negativization, while in the *Control* group microbiological negativization was observed in 36,4% (95% CI: [17–59]%) of patients, *p* < 0.01. Considering the side effects of the antibiotic therapy, 40% (95% CI: [12–73]%) of patients in the *Protocol* group developed AKI, while in the *Control* group only 4,5% (95% CI: [0,1–22]%) of patients, *p* = 0.01. All patients with AKI, in both groups, received renal replacement therapy and, in all patients, the renal function recovered before ICU discharge. No other relevant side effects related to antibiotic therapy were observed in both groups. All patients, 100% (95% CI: [69–100]%) in the *Protocol* group were discharged alive from ICU, while, in the *Control* group, 36.4% (95% CI: [17–59]%) of patients survived, *p* < 0.01, Table 3.

**TABLE 3:** Outcomes in the study population.

Outcomes	Protocol* (*n* = 10)	Control° (*n* = 22)	p-value**
Negativization, n (%)	10 (100)	8 (36.4)	<0.01
Complication – AKI, n (%)	4 (40)	1 (4,5)	0.01
ICU Survivors, n (%)	10 (100)	8 (36.4)	<0.01

*Protocol = colistin 9 million IU + 4.5 million IU every 12 h, intravenous tigecycline 100 mg every 12 h, intravenous ampicillin/sulbactam 12 gr per day and inhaled colistin 3 million IU every 6 h.

*°Control = nebulized and intravenous colistin alone or combined with an antibiotic of another class.*

**Chi-squared test or Fisher’s exact test, as appropriate. AKI = acute kidney injury.

## Discussion

The present brief report aimed to retrospectively describe the evolution of the superinfection from *PDR Acinetobacter baumannii* in patients with SARS-CoV-2 infection admitted to ICU and assess the incidence of negativization between patients treated with the combination of at least three antibiotics, according to the protocol applied in our ICU, and those who received a combination of two antibiotics. Our study shows that all patients in the *Protocol* group had microbiological negativization together with the clinical resolution of the infection and all of them were discharged alive from ICU. Considering the side effects of the antibiotic therapy, the patients in the *Protocol* group had a significantly higher incidence of AKI, which was managed in all cases with renal replacement therapy. However, the renal function recovered without sequelae in all patients before ICU discharge. Regarding the outcome, it is important to mention that the causes of death of patients in the *Control* group were not exclusively related to the complications of the *PDR Acinetobacter baumannii* superinfection. In fact, this study was focused on this single specific infection and the impact of this treatment protocol on microbiological negativization. No other co-infections, as well as other possible complications, were considered and the study itself was not designed to assess a cause-effect relationship with the outcome. However, considering the impact of this superinfection, the fact that all patients in the *Protocol* group survived was important to point out.

*Carbapenem-Resistant Acinetobacter baumannii*, as well as *Enterobacterales* and *Pseudomonas aeruginosa* resistant to carbapenems, were first on the WHO’s list of resistant bacteria for 2016-2017 as they threaten public health globally (27). In particular, among the 12000 annual infections in the United States, more than 60% of them were caused by *MDR Acinetobacter baumannii*, as remarked by the American CDC report in 2013 (28). The management of *MDR Acinetobacter baumannii* is currently based on carbapenems if the isolated microorganisms show susceptibility to this antibiotics class (17). With regards to *XDR Acinetobacter baumannii*, it is associated with a mortality rate higher than 50% (29, 30). Its recommended treatment consists of polymyxins and tigecycline. Whether colistin alone or in a combined therapy gives advantages or not, is still debatable (17, 30). As regards tigecycline, although standard doses did not seem to have an effect, high-dose tigecycline, defined as a loading dose of 200 mg followed by 100 mg every 12 h, lead to better results in terms of outcome (31).

A recent metanalysis by *Jung et al.*, regarding *MDR/XDR Acinetobacter baumannii*, showed that sulbactam, both at a normal and at a high dose, had the best survival benefit. Fosfomycin and colistin came second, followed by a combination of colistin given both by inhalation and intravenously, while monotherapy with high-dose of tigecycline and colistin came last (32). Only sulbactam showed activity against *Acinetobacter baumannii* but in most European countries, such as Greece and Italy, the only available combination is ampicillin/sulbactam (33). As *Acinetobacter baumannii* becomes *Pan-Drug Resistant*, treatment options significantly decrease in number. The problem of *Pan-Drug Resistant* Gram-negative bacteria is increasing worldwide, but the management of the *PDR Acinetobacter baumannii* infections is particularly hard (21). *Karakonstantis et al.*, in their cohort study, showed that in-hospital mortality is significantly higher in patients with *PDR Acinetobacter baumannii* infections compared to those with *PDR Acinetobacter baumannii* colonization (34). Moreover, typically affecting patients with critical illness, multimorbidity, and exposure to invasive procedures, the *PDR Acinetobacter baumannii* infections considerably prolong the length of hospitalization (34). To cope with the lack of effective treatments available, several studies have been performed to establish the effectiveness of current options available such as ceftazidime/avibactam and ceftolozane/tazobactam, but they did not show any significant advantages (35). Following this, if some data regarding the effectiveness of antibiotics against *XDR Acinetobacter baumannii* exist, no clinical data are available for *PDR Acinetobacter baumannii* (36–38). For this reason and given the difficulty in treating *PDR Acinetobacter baumannii*, *Assimakopoulos et al.* used a combination therapy, which seemed to have promising results *in vitro*. By administering colistin both nebulized and intravenous with high-dose tigecycline and high-dose ampicillin/sulbactam, they demonstrated a high rate of clinical response and the hitherto highest percentage of survival at 28 days (90%) (22).

Nonetheless, we must mention one of the newest cephalosporins, cefiderocol, which was inserted into the list of antimicrobials suitable for *MDR* Gram-negative infections, mainly for *MDR Acinetobacter baumannii* (39). As *Bassetti et al.* remarked in their review, in Europe, it has been used since 2020, while in the United States it was already approved in 2019 (39). Given its high costs and its initial no-refunds policy in Italy, it was not used routinely. The Italian Drug Agency (AIFA) did not approve its refundability until June 2021 and only for patients with limited or no further options of treatment. Nevertheless, cefiderocol showed advantageous clinical cure rates compared to the best available therapy in Gram-negative pneumonia caused by carbapenem-resistant Enterobacteriaceae, complicated urinary tract infections, bloodstream infections, and sepsis. Despite this, the all-cause mortality was found higher in patients treated with cefiderocol (39). Therefore, its use is restricted to those aged equal to or more than 18 years old with no other options (39).

Our study has some limitations. First of all, the retrospective design of the study does not allow to control for all confounding factors. Moreover, our attention, as mentioned above, was focused on a single infection and the impact of the treatment protocol, applied in our ICU, on microbiological negativization: we did not collect data about other possible co-infections. Regarding the side effects of antibiotic therapy, it was difficult to assess and report the exact incidence of the neurotoxicity of colistin. It is established that colistin, interacting with neurons, can cause a wide spectrum of neurological manifestations, such as peripheral and orofacial paresthesias, visual disturbances, vertigo, mental confusion, ataxia, and seizures (40). All these manifestations are difficult to assess in patients sedated and intubated in ICU. Furthermore, it is now known that the SARS-CoV-2 infection itself can lead to neurological effects (41) as well as hospitalization in ICU, which is related to the “critical illness polyneuropathy” (40). Furthermore, to date, we have not yet collected the data on COVID-19 patients, admitted to our ICU in a period following the study population, who developed *PDR Acinetobacter baumannii* superinfection, treated with cefiderocol. It may be useful to compare the two treatment strategies in terms of effectiveness and side effects.

## Conclusion

Our brief report shows microbiological negativization as well as the clinical resolution of the *PDR Acinetobacter baumannii* superinfection in all patients treated with the combination therapy of nebulized and intravenous colistin, high-dose tigecycline, and high-dose ampicillin/sulbactam. This combination of antibiotics seems to be a useful alternative to eradicate *PDR Acinetobacter baumannii* when cefiderocol is not easily accessible or may fail therapeutically.

## Data Availability Statement

The raw data supporting the conclusions of this article will be made available by the authors, without undue reservation.

## Ethics Statement

The studies involving human participants were reviewed and approved by Comitato Etico Regionale delle Marche – Azienda Ospedaliero Universitaria “Ospedali Riuniti,” Ancona. The ethics committee waived the requirement of written informed consent for participation.

## Author Contributions

EC collected the data, performed the statistical analysis, interpreted the data, and drafted the manuscript. EB contributed to drafting the manuscript and reviewing the literature. SV and RG collected the data. RD contributed to drafting and revising the manuscript. AC, ED, and CS participated in the interpretation of the results. VG, SP, and BM revised the manuscript. AD and EA designed the study, participated in the statistical analysis and interpretation of the data, and revised the manuscript. All authors approved the submitted version of the manuscript and agreed both to be personally accountable for the author’s contributions and to ensure that questions related to the accuracy or integrity of any part of the work, even ones in which the author was not personally involved, are appropriately investigated, resolved, and the resolution documented in the literature, and read and approved the final manuscript.

## Conflict of Interest

The authors declare that the research was conducted in the absence of any commercial or financial relationships that could be construed as a potential conflict of interest.

## Publisher’s Note

All claims expressed in this article are solely those of the authors and do not necessarily represent those of their affiliated organizations, or those of the publisher, the editors and the reviewers. Any product that may be evaluated in this article, or claim that may be made by its manufacturer, is not guaranteed or endorsed by the publisher.

## Abbreviations

COVID-19: COronaVIrusDisease-19
ICU: Intensive Care Unit
SARS-CoV-2: Severe Acute Respiratory Syndrome CoronaVirus-2
VAP: Ventilator Associated Pneumonia
MDR: Multi Drug-Resistant
XDR: eXtensively Drug-Resistant
PDR: Pan Drug-Resistant
SOFA: Sequential Organ Failure Assessment
AKI: Acute Kidney Injury
MIC: Minimum Inhibitory Concentration.
